# pam: An R Package for Fast and Efficient Processing of Pulse‐Amplitude Modulation Data

**DOI:** 10.1002/ece3.73400

**Published:** 2026-04-20

**Authors:** Julien Böhm, Philipp Schrag

**Affiliations:** ^1^ Department of Aquatic Ecology, Institute of Biosciences University of Rostock Rostock MV Germany; ^2^ Lindenberg im Allgäu Bavaria Germany

**Keywords:** automated data processing, PAM, photosynthesis, R, rapid light curve, WALZ

## Abstract

Rapid light curves recorded via the pulse‐amplitude modulation (PAM) technique are widely used to characterize photosynthesis, enabling the determination of key photosynthetic parameters. However, deriving these kinetic parameters from raw data requires fitting to regression models, a process traditionally involving laborious and error‐prone manual steps. Our R package pam streamlines this process by automating regression analysis, enabling fast and reproducible processing of large datasets. It provides the models of Vollenweider (1965), Platt et al. (1980), Eilers and Peeters (1988) and Walsby (1997). To demonstrate the functionality of the package, we present a workflow including data reading, model fitting, data validation, and data export using a dataset of 20 files and compare the results with those obtained from the built‐in WALZ Solver and the Excel Solver add‐on. The workflow successfully processed all data, produced reliable results, and executed in less than 20 s, making it several times faster than traditional approaches, such as using the Excel Solver add‐on. This R package and its source code are freely available on GitHub at https://github.com/biotoolbox/pam and CRAN at https://cran.r‐project.org/web/packages/pam/index.html.

## Graphical abstract

The R package pam provides a fast and efficient pipeline for processing light curves recorded via the pulse‐amplitude modulation (PAM) technique. By integrating data import, model fitting, validation, and export, it enables robust and reproducible estimation of key photosynthetic parameters within seconds. This open‐source tool accelerates analyses while minimizing manual effort and human error.

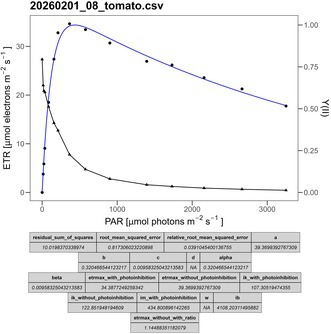

## 1 Introduction

Rapid light curves measured using the PAM (pulse‐amplitude modulation) technique depict the relationship between light intensity (e.g., in μmol photons m^−2^ s^−1^) and the rate of photosynthesis (e.g., electron transport rates) and are an established tool for assessing how efficiently an organism converts light into chemical energy (Schreiber 2004). They enable the determination of key kinetic parameters such as the maximum electron transport rate (ETR_max_), the light intensity at the transition point from light limitation to light saturation (I_k_), or the efficiency of photosynthesis under low light conditions (α (alpha)) and high light conditions (β (beta)) (Schreiber 2004; Ralph and Gademann 2005), which are critical for assessing photosynthetic performance, stress tolerance, or responses to environmental conditions (Ralph and Gademann 2005). However, to derive these parameters from the measurements, they need to be fitted to regression models. Over the past decades, several models have been developed for modeling photosynthesis‐light curves (e.g., Vollenweider 1965; Jassby and Platt 1976; Platt et al. 1980; Eilers and Peeters 1988; Walsby 1997) and adapted for analyzing PAM data (e.g., Romoth et al. 2019; Heise et al. 2024; Böhm et al. 2025; Maidel and Schubert 2026). Their application necessitates numerical approximations. These regressions are often performed by using a solver add‐on in Excel (Frontline Systems Inc. 2009) or built‐in functions of software that operates the PAM device. Unfortunately, this approach is time‐consuming, error‐prone, and lacks clarity when applied to large datasets due to the multiple manual steps, leading to reduced reproducibility—a general concern in scientific studies (Baker 2016). The R package pam provides a solution by optimizing the workflow. It allows for the automated processing of large datasets, including data import, regression analysis, fit evaluation, and the export of results for further processing, ensuring fast, reproducible, and transparent analyses through open‐source code, thorough documentation, automated tests, and a clear audit trail. While general implementations for gas exchange and photosynthesis models already exist in R (Duursma 2015; Stinziano et al. 2021), our package provides the first end‐to‐end pipeline specifically tailored to PAM measurements. To demonstrate its applicability, we present a workflow utilizing a large experimental dataset of 20 measurements and compare the results to those obtained with the built‐in WALZ Solver tool (Software: Dual PAM, Heinz WALZ GmbH, Christof Klughammer, Effeltrich, Germany) and the Excel Solver add‐on. For this purpose, we use the models of Platt et al. (1980) and Eilers and Peeters (1988), as these are the only models available in the built‐in WALZ Solver tool.

## 2 Implementation

### 2.1 Installation and Usage

The package pam (2.1.1) was developed under R Version 4.4.2 (R Core Team 2024) and can be installed either from CRAN (install.packages(“pam”)) or from GitHub (remotes::install_github(“biotoolbox/pam”, subdir = “src”)). Within the pam package, several R packages are used. Data handling is performed using data.table (Barrett et al. 2026), and non‐linear regression is carried out using minpack.lm (Elzhov et al. 2023), which implements the Levenberg–Marquardt algorithm and ensures robust optimization. The calculation of evaluation metrics is performed using Metrics (Hamner and Frasco 2018). Visualization relies on ggplot2 (Wickham 2016), cowplot (Wilke 2025), gridExtra (Auguie 2017) and ggthemes (Arnold 2025). Four regression models are available for analysis: Vollenweider (1965), Platt et al. (1980), Eilers and Peeters (1988) and Walsby (1997). All functions and their usage are presented in Table 1. Table 2 provides an overview of all parameters and their respective meanings. The source code is available on GitHub: https://github.com/biotoolbox/pam, CRAN: https://cran.r‐project.org/web/packages/pam/index.html and Zenodo: https://zenodo.org/records/19567394.

**TABLE 1:** Function overview of key components in the pam package. Sorted according to user logic, starting with the read functions and ending with the export functions.

Function	Usage
read_universal_data()	Imports raw CSV data from a standardized CSV file
read_dual_pam_data()	Imports raw CSV data from Dual‐PAM instrument
read_junior_pam_data()	Imports raw CSV data from Junior‐PAM instrument
compare_regression_models_ETR_I()	Compares regression models for ETR I data
compare_regression_models_ETR_II()	Compares regression models for ETR II data
eilers_peeters_generate_regression_ETR_I()	Fits a regression model for ETR I following Eilers and Peeters (1988)
eilers_peeters_generate_regression_ETR_II()	Fits a regression model for ETR II following Eilers and Peeters (1988)
eilers_peeters_modified()	Modifies the Eilers and Peeters (1988) model by standardizing parameter names
platt_generate_regression_ETR_I()	Fits a regression model for ETR I based on Platt et al. (1980)
platt_generate_regression_ETR_II()	Fits a regression model for ETR II based on Platt et al. (1980)
platt_modified()	Extends the Platt et al. (1980) model by adding parameters and standardizing names
vollenweider_generate_regression_ETR_I()	Fits a regression model for ETR I following Vollenweider (1965)
vollenweider_generate_regression_ETR_II()	Fits a regression model for ETR II following Vollenweider (1965)
vollenweider_modified()	Extends the Vollenweider (1965) model by adding parameters and standardizing names
walsby_generate_regression_ETR_I()	Fits an ETR I regression model based on Walsby (1997), with the respiration term removed and naming conventions as in Romoth et al. (2019)
walsby_generate_regression_ETR_II()	Fits an ETR II regression model based on Walsby (1997), with the respiration term removed and naming conventions as in Romoth et al. (2019)
walsby_modified()	Extends the Walsby (1997) model by adding parameters and standardizing names
combo_plot_control()	Generates a combined plot of ETR data showing fits from different regression models along with summary tables
plot_control()	Creates control plot and summary table for a given model and dataset
write_model_result_csv()	Exports intermediate table data, regression results, and model parameters to separate CSV files for further analysis

**TABLE 2:** Publication‐accurate naming and respective modified naming with additional calculations not included or not explicitly outlined in the original publication.

Modified	Eilers and Peeters	Platt	Walsby	Vollenweider	Explanation
residual_sum_of_squares	residual_sum_of_squares *	residual_sum_of_squares *	residual_sum_of_squares *	residual_sum_of_squares *	Deviation between the data points and the respective regression, expressed as the sum of squared residuals
root_mean_squared_error	root_mean_squared_error *	root_mean_squared_error *	root_mean_squared_error *	root_mean_squared_error *	Deviation between the data points and the respective regression, expressed as the root mean squared error
relative_root_mean_squared_error	relative_root_mean_squared_error *	relative_root_mean_squared_error *	relative_root_mean_squared_error *	relative_root_mean_squared_error *	Deviation between the data points and the respective regression, expressed as the relative root mean squared error, normalized by the mean
a	a	ps	etr_max	pmax	Regression model parameter; may be a direct or intermediate parameter
b	b	alpha	alpha	a	Regression model parameter; may be a direct or intermediate parameter
c	c	beta	beta	alpha	Regression model parameter; may be a direct or intermediate parameter
d	NA	NA	NA	n	Regression model parameter; may be a direct or intermediate parameter
alpha	s	alpha	alpha *	real_alpha *	Initial slope of the curve (electrons/photons); efficiency of photosynthesis under low light conditions
beta	NA	beta	beta	NA	Index for photoinhibition
etrmax_with_photoinhibition	pm	pm	etrmax_with_photoinhibition *	popt	Maximum electron transport rate under the effect of photoinhibition (μmol electrons m^−2^ s^−1^)
etrmax_without_photoinhibition	NA	ps	etr_max	pmax	Maximum electron transport rate in the absence of photoinhibition (μmol electrons m^−2^ s^−1^)
ik_with_photoinhibition	ik	ik	ik_with_photoinhibition *	iik	PAR at the transition point from light limitation to light saturation under the effect of photoinhibition (μmol photons m^−2^ s^−1^)
ik_without_photoinhibition	NA	is	ik_without_photoinhibition *	ik	PAR at the transition point from light limitation to light saturation in the absence of photoinhibition (μmol photons m^−2^ s^−1^)
im_with_photoinhibition	im	im	im_with_photoinhibition *	im_with_photoinhibition *	PAR at which the maximum electron transport rate under photoinhibition is reached (μmol photons m^−2^ s^−1^)
w	w	NA	NA	NA	Quantifies the sharpness of the peak of the curve
ib	NA	ib	NA	NA	PAR at which the light curve attains 1/e of the maximum electron transport rate in the absence of photoinhibition (μmol photons m^−2^ s^−1^)
etrmax_without_with_ratio	NA	etrmax_without_with_ratio *	etrmax_without_with_ratio *	pmax_popt_and_ik_iik_ratio	Ratio of maximum electron transport rate without and with photoinhibition

*Note:* An asterisk (*) indicates a parameter not included or not explicitly outlined in the original publication, while NA means it is not available.

### 2.2 Example Workflow

The raw data in CSV format can be imported using one of the read functions (e.g., read_dual_pam_data()), which offers options to adjust the photosystem ratio and the ETR factor. Measurement points unrelated to the light curve, such as recovery measurements, can be excluded during this step. The goodness of the fit between models can be assessed by using the compare_regression_models_ETR_II() function, which compares the residual sum of squares of the models. After selecting a model, the regression is fitted, and parameters are computed using the respective model function, for example, platt_generate_regression_ETR_II(). Start parameters for the regression have a default value but can also be adjusted. Additionally, the platt_modified() function ensures a consistent parameter naming scheme across models and incorporates parameters originally introduced in other models. The quality of the regression can be visually evaluated using the plot_control() function. Results can be exported using write_model_result_csv() for further analysis. This function generates three CSV files: one containing the intermediate table data, one with the regression data, and one with the computed parameters such as ETR_max_. The package also supports processing Photosystem I data. While the functions are designed for single files, the workflow for multiple files can be efficiently automated using a loop. Documentation for each function can be accessed by typing ?function_name (e.g., ?write_model_result_csv()) in the R console and is also available on GitHub.

The dataset used here consists of 20 files, measured with a dual PAM chlorophyll fluorometer/absorption spectrometer (DUAL‐PAM‐100, Heinz Walz GmbH, Effeltrich, Germany). The scripts used to derive the parameters for the solver comparison are presented for the Platt et al. (1980) model in Listing 1 and for the Eilers and Peeters (1988) model in Listing S1.

**LISTING 1:**
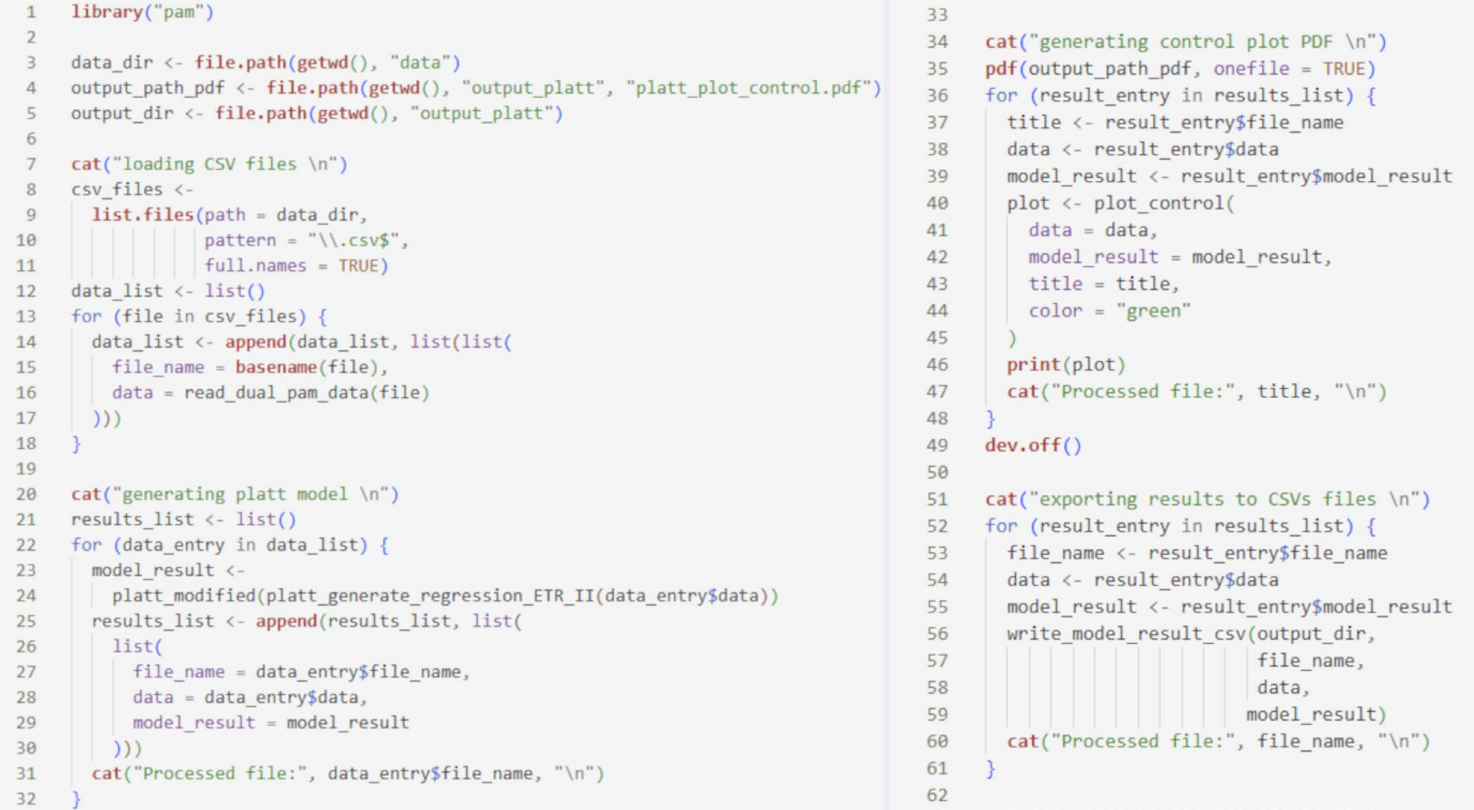
Workflow for processing multiple files using the Platt et al. (1980) model: CSV files are first read, regression models are fitted, control plots are generated, and the results are subsequently exported as CSV files.

## 3 Results and Discussion

**FIGURE 1:**
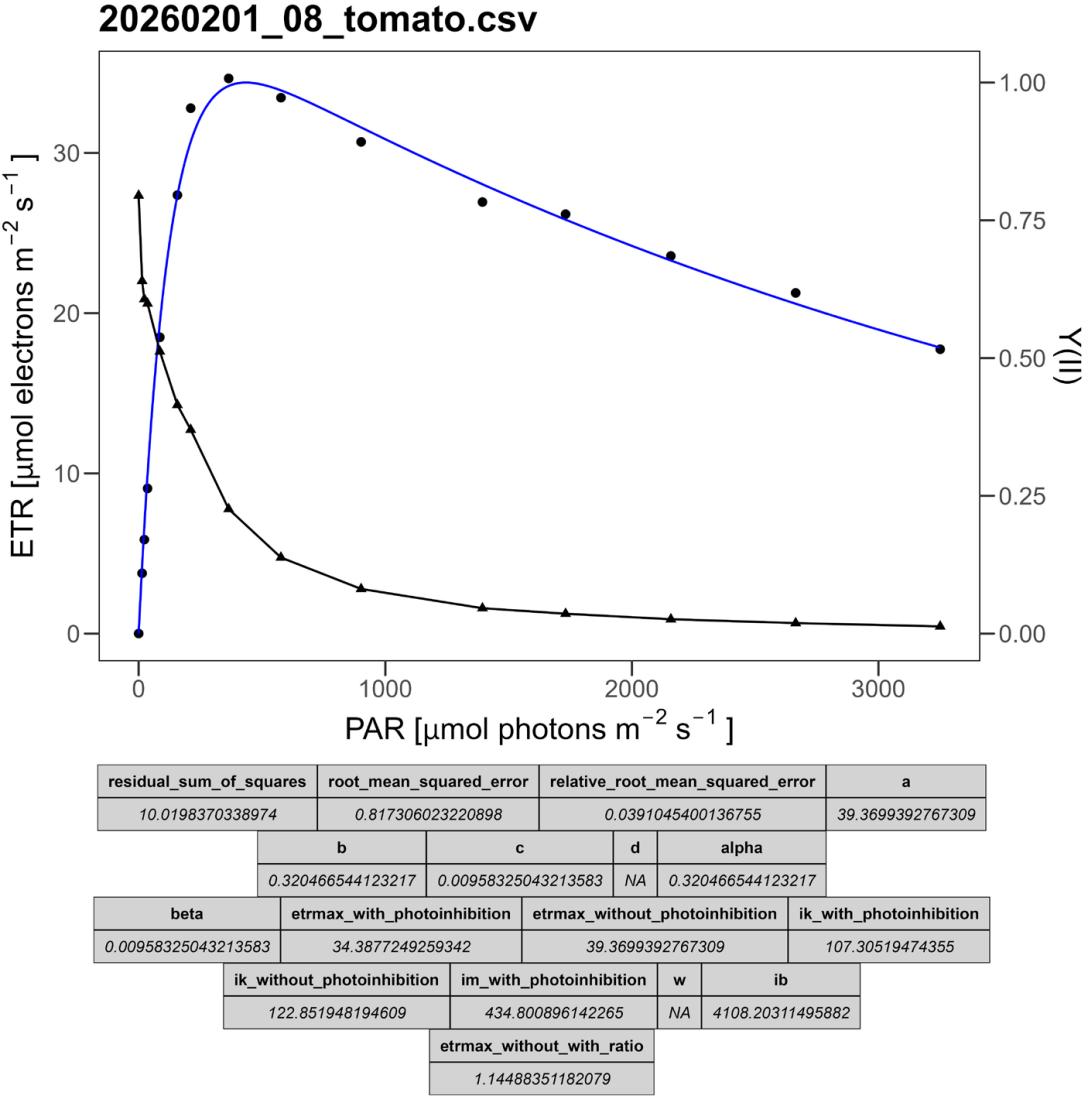
Example control plot for regression evaluation. The control plot displays Y(II) (black triangles), the calculated ETR (black dots), and the corresponding regression using the model of Platt et al. (1980) (blue line). The table below lists all parameters in the modified layout. “NA” indicates that the parameter is either not available for the model or could not be determined.

The scripts using Platt et al. (1980) and Eilers and Peeters (1988) models executed in 9.1 s and 8.7 s (median of 10 runs), respectively, for a total runtime of under 20 s on a PC with a Ryzen 5 5600X (AMD, Santa Clara, USA). The duration may vary depending on the hardware used. In contrast, using a manual fitting workflow based on the solver add‐on in Excel required an experienced researcher approximately 45 min for both models. By running the scripts, a PDF file with control plots for each model was successfully generated. A visual inspection of all plots showed that both the fits and the determination of all parameters, such as ETR_max_, α, and I_k_, were successful. An example control plot from the dataset is shown in Figure 1. The results were then exported as a CSV file, enabling automated import for further processing and visualization. If a fit fails, an error message is returned, and we recommend restarting the script with different start parameter values.

**FIGURE 2:**
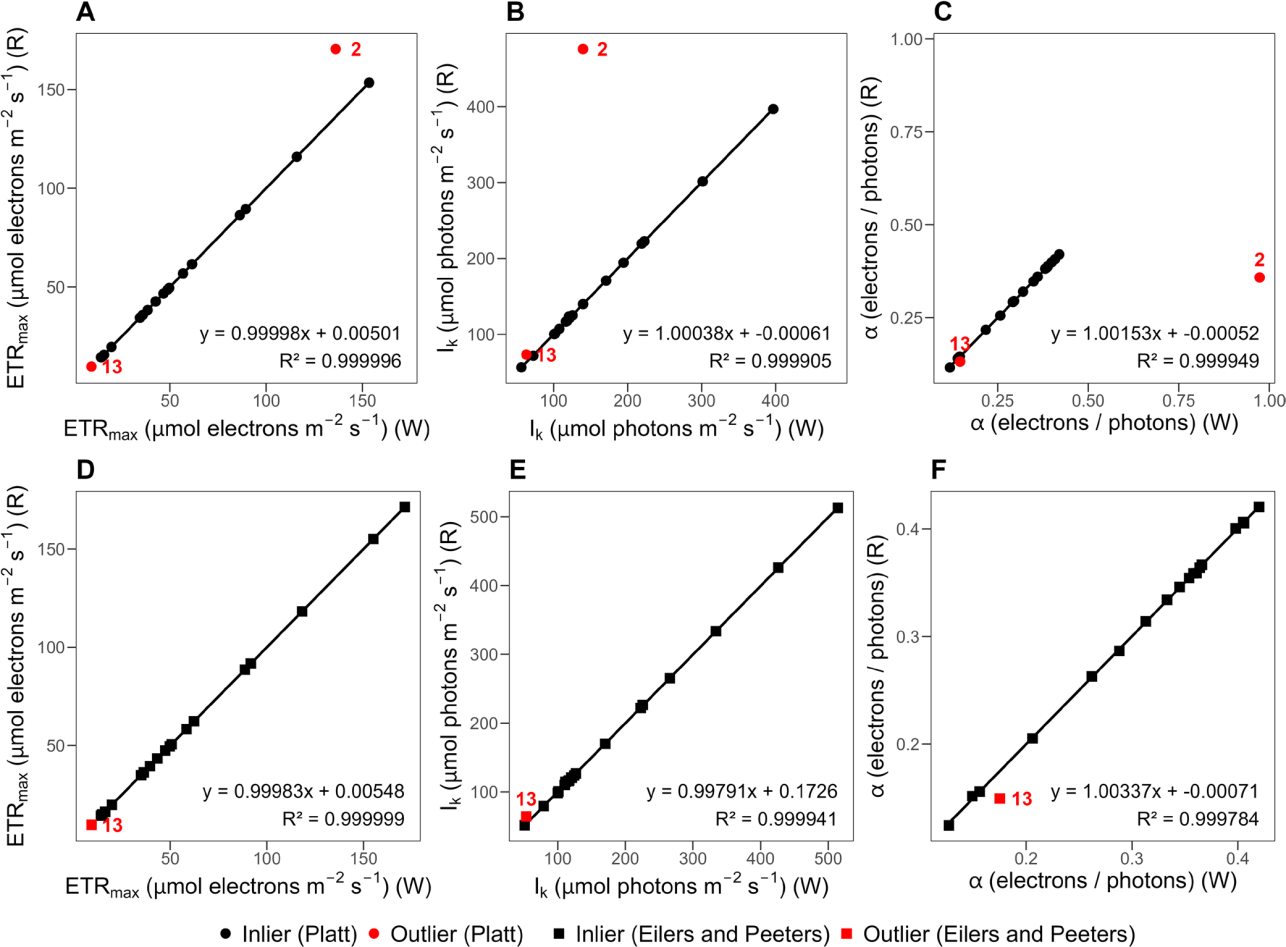
Comparison of the WALZ solver (W) and the pam package (R) for estimating the cardinal photosynthetic parameters ETR_max_, I_k_, and α, including photoinhibition, using the models of Platt et al. (1980) and Eilers and Peeters (1988). Panels (A–C) show results obtained with the Platt model and (D–F) results obtained with the Eilers and Peeters model. Linear regressions were fitted using inliers only. Outliers are defined as WALZ solver (W) values whose relative difference from the pam package (R) predictions exceeds 3%. The number above each outlier corresponds to the measurement number for verification. The regression equation and *R* ^2^ are shown in the lower right corner of each panel.

**FIGURE 3:**
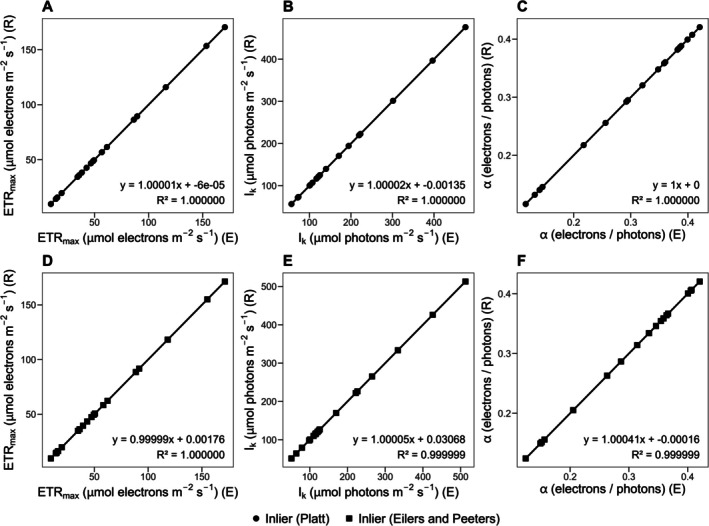
Comparison of the Excel Solver add‐on (E) and the pam package (R) for estimating the cardinal photosynthetic parameters ETR_max_, I_k_, and α, including photoinhibition, using the models of Platt et al. (1980) and Eilers and Peeters (1988). Panels (A–C) show results obtained with the Platt model and (D–F) results obtained with the Eilers and Peeters model. Linear regressions were fitted after testing for outliers; however, no outliers were detected. Outliers are defined as Excel Solver add‐on (E) values whose relative difference from the pam package (R) predictions exceeds 3%. The regression equation and *R* ^2^ are shown in the lower right corner of each panel.

Generated results were then validated against the WALZ solver (Figure 2) and the Excel solver (Figure 3). In comparison with the WALZ solver, the Platt et al. (1980) model yielded very similar results for 18 out of 20 measurements, as evidenced by linear regressions exhibiting slopes close to 1, relatively small intercepts, and *R* ^2^ > 0.9999. However, two outliers were detected, corresponding to measurements 2 and 13. A similar outcome was obtained for the Eilers and Peeters (1988) model, with one outlier identified in measurement 13. A closer inspection revealed that for measurement 2, the WALZ solver did not produced a proper result for the Platt model (Figure S1), whereas for measurement 13, the last light step yielded a Y(II) value of 0. This value does not appear to be displayed or processed in the WALZ interface, but it is present in the CSV export and is therefore handled by our package (Figure S2). In comparison with the Excel solver add‐on, no outliers were detected, and the slopes were close to 1 in all cases, with relatively small intercepts and *R* ^2^ > 0.999999. Overall, the comparisons confirm that the pam package produces reliable and reproducible results.

In summary, a fast, efficient and reproducible workflow has been demonstrated, requiring few manual steps, making it robust against human errors. While the package can already be used for regression of data from devices other than the Dual PAM and Junior PAM via the universal read function, which requires users to pre‐format their data into the expected structure, we plan to add additional device‐specific read functions to further extend the package's usability. Furthermore, future development plans include fitting non‐photochemical quenching (NPQ) kinetics and calculating yield ratios between Photosystem II and Photosystem I.

## Author Contributions

**Julien Böhm:** conceptualization (lead), methodology (equal), software (equal), writing – original draft (lead), writing – review and editing (lead). **Philipp Schrag:** conceptualization (supporting), methodology (equal), software (equal), writing – review and editing (supporting).

## Funding

The authors have nothing to report.

## Consent

All authors agreed to publish the present manuscript.

## Conflicts of Interest

The authors declare no conflicts of interest.

## Supporting information


**Figure S1:** Measurement 2: Comparison of fits using the Platt et al. (1980) model derived from the WALZ solver (red dashed line, A) and the pam package (green line, B).

**Figure S2:** Measurement 13: Comparison of fits using the Platt et al. (1980) model derived from the WALZ solver (red dashed line, B) and the pam package (green line, A), with the corresponding raw data shown in C.

**Listing S1.** Workflow for processing multiple files using the Eilers and Peeters (1988) model: CSV files are first read, regression models are fitted, control plots are generated, and the results are subsequently exported as CSV files.

## Data Availability

The pam R package, introduced in this study, is open‐source software and openly available on GitHub (https://github.com/biotoolbox/pam) and CRAN (https://cran.r‐project.org/web/packages/pam/). It is released under the GPL‐3.0 license. Version 2.1.1 (introduced here) is available on Zenodo as well (https://zenodo.org/records/19567394). Users are encouraged to report any issues or further questions via GitHub Issues (https://github.com/biotoolbox/pam/issues). Source code for additional calculations in this paper (e.g., the solver comparison) is available at https://github.com/biotoolbox/pam‐paper‐figures.
