# Supplementary figures and images for: pam: An R Package for Fast and Efficient Processing of Pulse‐Amplitude Modulation Data
Source: Ecol Evol. 2026 Apr 20;16(4):e73400. doi: 10.1002/ece3.73400 (PMC13095479; doi:10.1002/ece3.73400)

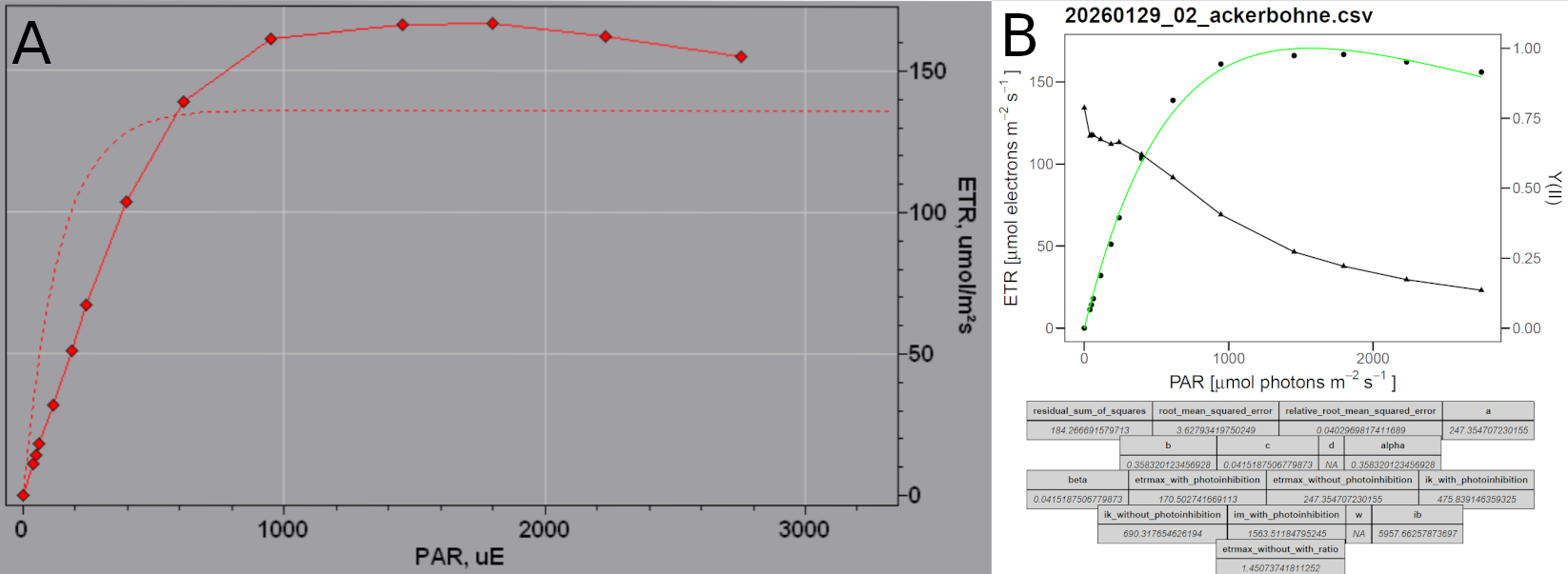

Supplement: Supplementary file 1 — Figure S1: Measurement 2: Comparison of fits using the Platt et al. (1980) model derived from the WALZ solver (red dashed line, A) and the pam package (green line, B). [file ECE3-16-e73400-s001.png]

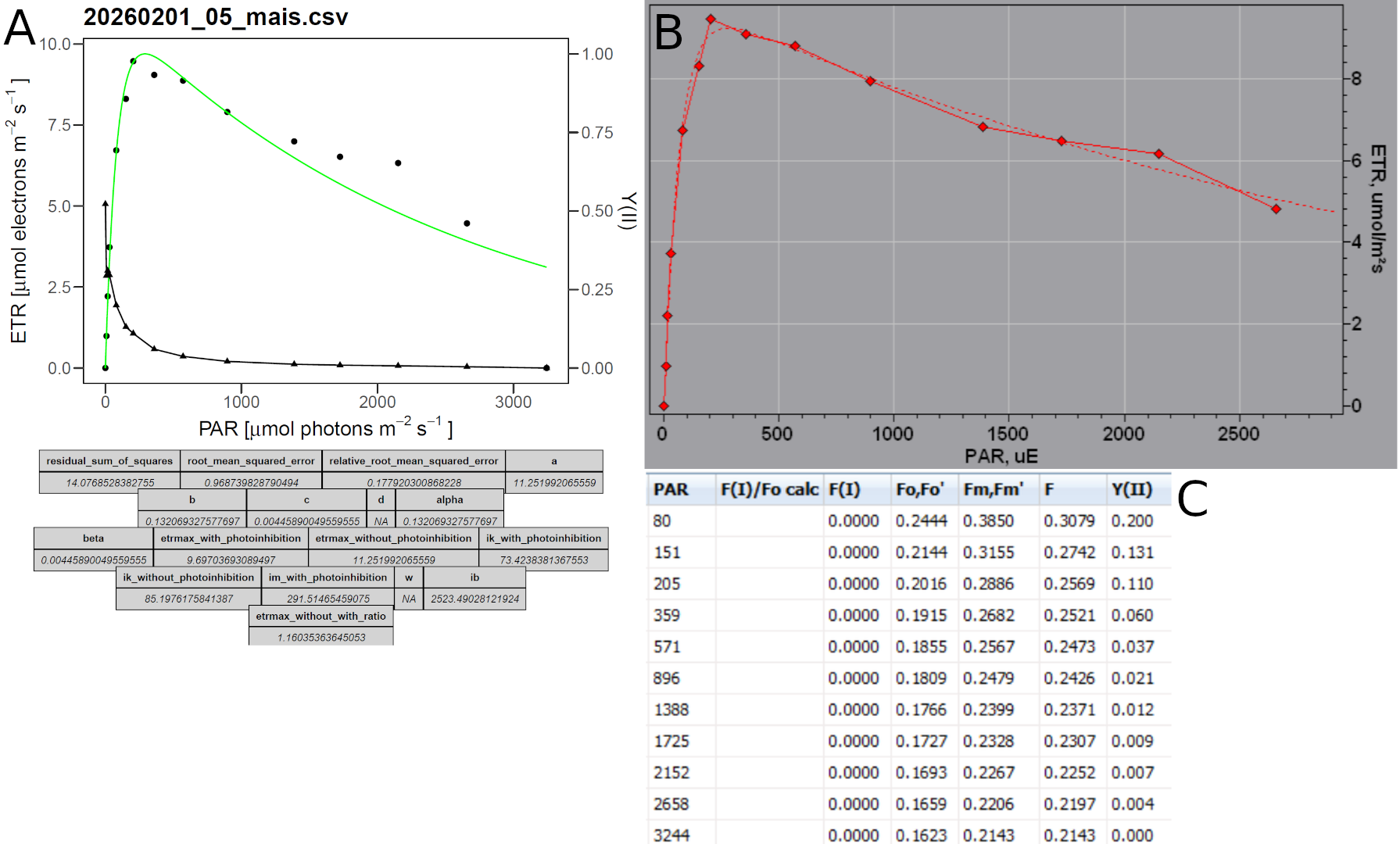

Supplement: Supplementary file 2 — Figure S2: Measurement 13: Comparison of fits using the Platt et al. (1980) model derived from the WALZ solver (red dashed line, B) and the pam package (green line, A), with the corresponding raw data shown in C. [file ECE3-16-e73400-s002.png]

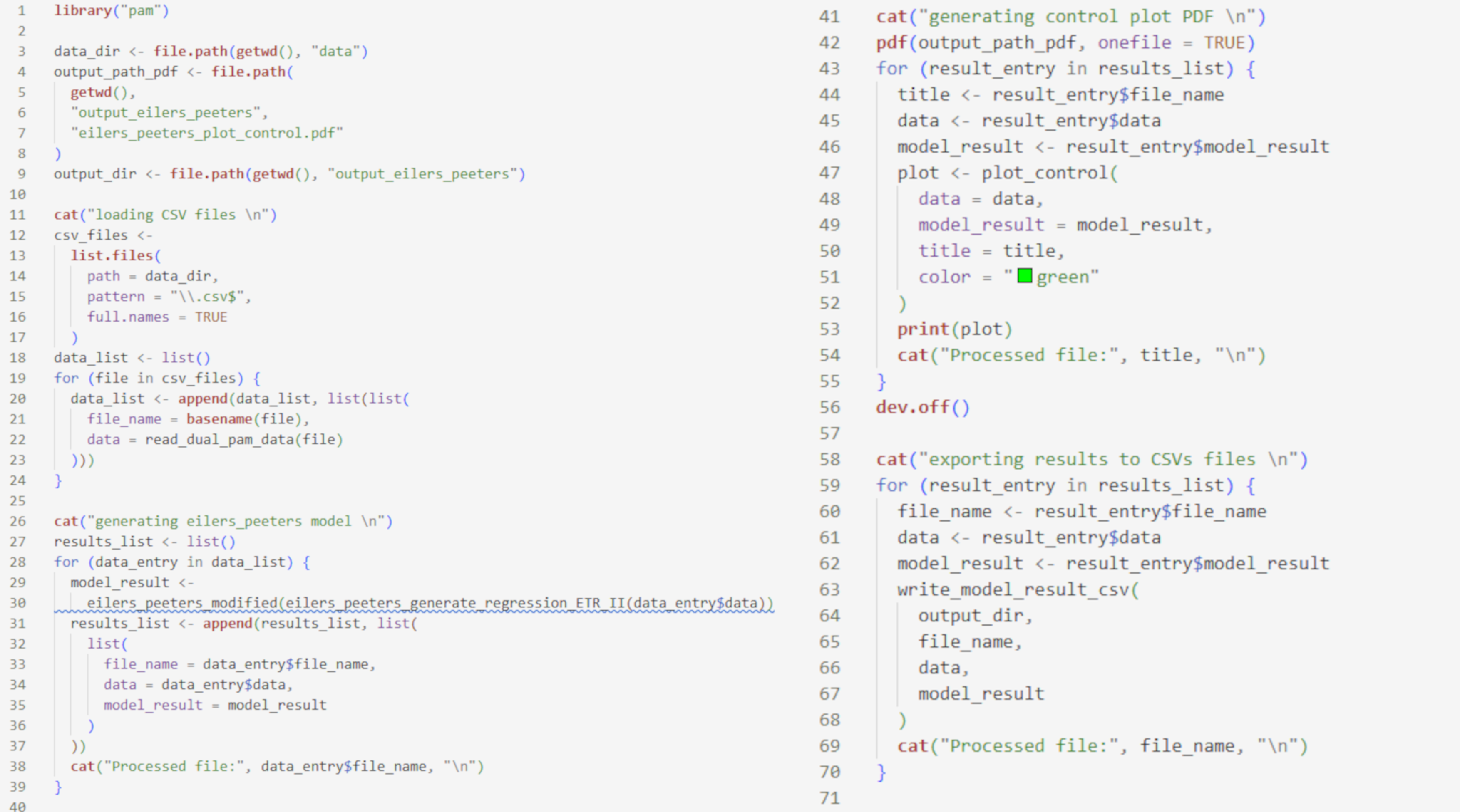

Supplement: Supplementary file 3 — Listing S1. Workflow for processing multiple files using the Eilers and Peeters (1988) model: CSV files are first read, regression models are fitted, control plots are generated, and the results are subsequently exported as CSV files. [file ECE3-16-e73400-s003.png]
